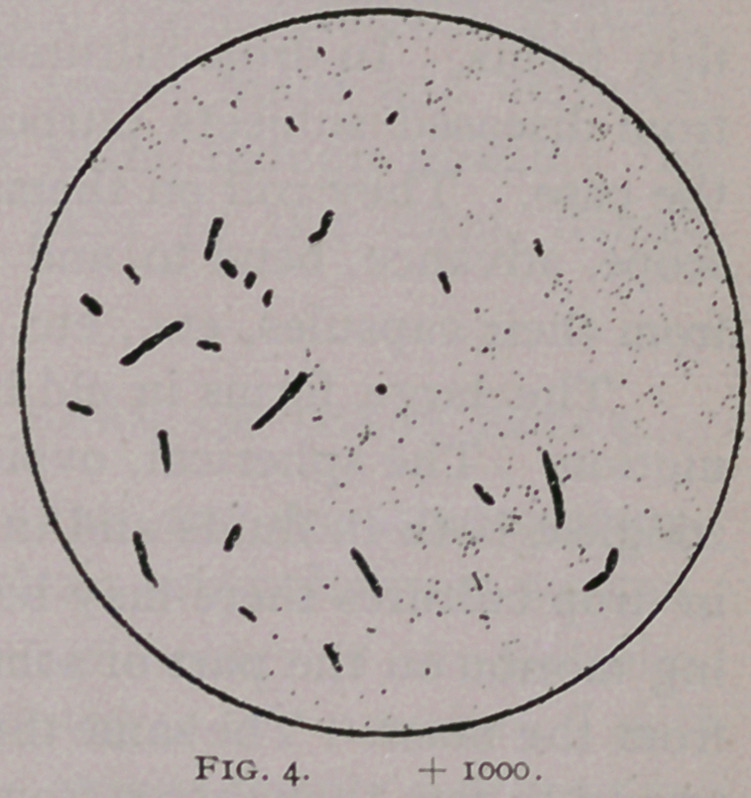# Texas Fever (Southern Cattle Fever, Etc.)

**Published:** 1890-08

**Authors:** Paul Paquin

**Affiliations:** State Veterinarian, Missouri


### TEXAS FEVER.

### (SOUTHERN CATTLE FEVER, ETC.)

By Paul Paquin,
*State Veterinarian, Missouri.*

(Concluded from Page 383.)

### THE GERM OF TEXAS FEVER.

A MALADY due to a germ is simply one produced by very small parasites; microscopic organisms termed germs, microbes, bacteria, etc., which grow in the body at the expense of the tissues, blood, etc., and thereby cause various kinds of disorders, such as fever, nervous phenomena and several other symptoms. Therefore the knowledge of the life of such germs is necessary to understand thoroughly the nature of the diseases that they cause, respectively. However, the explanation of such biological questions is of more interest to science than to practical men; and as we are still investigating doubtful points concerning the life of the germ of Texas fever, and as we have much material of more immediate utility to the masses, I will write this time as briefly as the problem allows on this particular point, upon which it is my intention to publish special articles, practical and scientific, some time in the future. As explained in the beginning of this bulletin, we have purposely abstained during our work from consulting any writer on Texas fever. Before writing this, however, I have carefully and conscientiously gone over the reports of the investigators who have studied Texas fever before us, and have compared the results with ours, in which we have much valuable positive and negative evidence.[1]

In regard to the priority in the discovery of the germ I have this to say: It seems that almost every investigator who has used the microscope has seen it in one or more of the stages of its development, but none have fully realized its significance. Dr.

[1] While preparing notes for this, Dr. F. S. Billings kindly forwarded to me, unsolicited, some slides made when he studied Texas fever. I can only mention this favor at this writing.

H. J. Detmers and Dr. D. E. Salmon have, at the time, attached some importance to the forms that they respectively noticed, and Dr. Detmers has, I am informed, always had faith in his findings. He deserves credit for his tenacity to a just cause. The Bureau of Animal Industry, however, seems to have abandoned whatever significance its officers have attached to the figure-8 germs that they found in the bile, and which really is one of the appearances of the microbe in question, for not long since Dr. Theobald Smith, of that section of the United States Department of Agriculture, published an interesting account of his latest researches, and claims that the disease is due to intra-globular forms not susceptible of cultivation, and that their effects are only on the red blood corpuscles which they destroy. He has not succeeded in inoculating the disease. I myself have seen the germ of Texas fever in spleens as far back as 1886, but I did not then appreciate its value. But Dr. Frank S. Billings has grasped and explained better than any the significance of at least one form of the germ. He has understood much that investigators before him misunderstood, and has foreseen largely the possibilities and properties of the parasite.

Judging from researches necessarily limited by time and circumstances, Dr. Billings concluded from his observations of the germs in tissues and culture, that in the mature form their longitudinal diameter is about twice that of their transverse, that they are very small, ovoid, and have rounded extremities with special affinity for stain. (Pages 75 and 76 of Bulletin of Agricultural Experiment Station of Nebraska. Nos. 7, 8, 9, 10, June, 1889.) It is true one finds the ovoid (and cocoid?) forms in the animal organisms and in cultures, but after investigating during two years a very large amount of animal tissues and fluids of various kinds, and after studying a great number of cultures, I believe that conclusion doubtful. The *small* ovoid seems only a period during the vegetation of the organism before complete maturity. From present indications I think the mature form is different and much larger, and perhaps the organism completes its life outside of the body. But I will let my readers judge for themselves. In analyzing the liver and spleen of infectious Southern cattle we may find two or three forms, *i. e.*, forms more or less ovoid which predominate and may be scattered, but are mostly grouped in masses in the *intimity*[1] of the tissues and corpuscles; a few short rods around which may occasionally be seen an areola (a light shade), and long large rod forms, sparsely distributed here and there. If cultures are made from such material the ovoid forms elongate, become surrounded by a gelatinous capsule or areola, and break off into at least two short rods, or double and single bodies mostly ovoid in appearance, and they may be seen with a capsule. By special processes of staining it is possible to obtain the capsule and all in same body. It is also possible to get all these forms on one slide and have them all at once under the microscope; observe Fig. 1.[2] There may be seen the single and double (cocoid and ovoid) bodies, elongating forms, large rods, some showing the surrounding capsule, etc. This illustration is exact, and is from an absolutely pure and inoculable culture made from the bile of an infectious Arkansas cow—bile with which we afterwards produced Texas fever. It shows at a glance, and better than I could describe, the various forms which this germ assumes during its growth, and made apparent by staining. And now, if you will study all the figures, you will perhaps form an idea as to the adult form. In the figure from a photograph of bile with the bacteria that it contained, will be seen forms which *perhaps* represent the truly adult stage as in the outer world. It is comparatively easy to get all the shapes and appearances that I have mentioned. To observe them all, one only has to study very carefully, by safe and sure bacterial methods and *for months*, the vegetation of the germs in different culture media and under various conditions, whether the origin was from Southern cattle of infectious grounds, or from Northern stock suffering from Texas fever, or from any reliable source. Perhaps he will be confused for a time in finding so many shapes and sizes, but presently he will get into the right groove and will be able to trace the origin of every one, especially if drop cultures, and drops of blood (under proper

[1] This word intimity is perhaps not used in the English language, but it has a correspondent in French, and means about this: mingled closely within. P. P.

[2] After writing the text, I found that a pure culture from blood of an affected Missouri native bovine presented exactly the same appearances under the microscope as that from this culture from the bile of an Arkansas cow, consequently I concluded to use but one cut for both of these cultures. Unfortunately the necessary changes or explanations in the text was forgotten. Hence the conflicting statements here, with explanations accompanying Fig. 1. This plate is really from a blood culture.

care) of infected stock are patiently and long watched. The long or large germs found in fresh organs disappear gradually, and the smaller ones only are found in preserved specimens, especially those kept in alcohol.

If one immerses in pure glycerine a fresh piece of liver of infectious Southern cattle, or of any Northern animal with the fever, in a month or more there may be seen on its surface fine white specks that gradually develop until they are roundish bodies as large as the head of a pin or larger. A section through the tissue will reveal the same condition of things within the organ. These granules (and neighboring substances) contain crystals, and living inoculable germs of various appearances, but they are seemingly those of Texas fever. A way to find several forms of the germ, is to fill a sterilized pipette of bile from the gall bladder of an infected Southern animal, or better a Northern with the fever, seal it and let it stand for some weeks or months; gradually whitish granules and flakes will be deposited as in the liver and forms may be noticed. Occasionally many appear stained brown, perhaps by the coloring matter of the bile. (Chemical analysis of these deposits are now under way by Prof. Schweitzer.) This color and the crystals mostly disappear in contact with acids. These substances are decidedly more prolific and pronounced in bile from an acute case of fever.

It may be objected that there was some "*post-mortem history*" connected with our germs, our cultures, the liver, bile and other specimens from which we form opinions. The fact is we have taken the greatest possible care to meet this convenient objection, and we have had, generally speaking, the same results from infectious Southern cattle *killed* in Texas, in the Indian Territory, in Arkansas, those brought here and *killed* purposely; from natives with the fever *killed* purposely, or immediately after death had occurred, and from inoculated cattle, pigs, and other animals. Identical cultures were obtained from *infectious* animals of the South, and *diseased cases* in the North, in the field and at the laboratory. As to those granules, as will be seen further, we have succeeded in inoculating them to cattle, and other animals, and produced seemingly the forms of Texas fever germs.[1] We have not

[1] I do not yet consider these inoculations positive evidence, however. P. P.

had the above results with liver and bile of Northern cattle *not* suffering from Texas fever, or exposed to its virus. As hinted, perhaps the parasite passes only a phase of its life within the animal body, and completes it outside, as was suggested by Dr. Theobald Smith, of the Bureau of Animal Industry, in the article already referred to and entitled "Preliminary Observations of the Micro-Organism of Texas Fever," contributed to the *Medical News*, Philadelphia, December 21st, 1889. In the kidneys, lymphatic glands, blood and urine, the germs are usually in some small forms. As Dr. Smith states, the red blood corpuscles become affected. In cases of fever in Northern stock, small forms adhere to the blood corpuscles, penetrate into them, and cause them to appear crenelated, irregular in shape, lumpy, knotty from the parasitic bodies within; perforated, distorted in fact. They seem to perish so rapidly, that in a few hours or in a few days at most, the blood has lost much of its solid elements, becomes more watery, and hæmogloblin passes into the urine.[1] But it is incomparably less severe in Southern stock. In their blood we find the same germs in lesser number, and the blood corpuscles are in part more or less diseased too, but they resist, as is evidenced by the apparently good health of the subjects; however, their livers and certain other organs are full of the parasites. The tissues and blood of an infectious Southern animal are much like the tissues and blood of a case produced by inoculation of *most of ordinary cultures*, or other mild virus. Dr. Smith, in the article quoted, formulates the following definition of Texas fever. "It is essentially a blood disease. There is a continous or paroxysmal destruction of red-blood corpuscles due to an intra-globular parasite, and the disease results mainly from the incapacity of the internal organs, primarily the liver, secondarily the spleen and kidneys, to transform and remove the waste products resulting from such destruction. . . . ."

From our work, I could scarcely admit such an exclusive definition. The germs being present outside of blood corpuscles, in the liver, spleen, lymph glands, kidneys, *as well as* in the blood of Southern infectious cattle and diseased Northern stock, and growing more or less in all of these organs and fluids, indicate a modification of this conception. Since around, on, and in the

[1] The urine becomes bloody in appearance.

blood corpuscles, as Dr. Smith has well said, are found almost only cocoid and ovoid bodies, single or in pairs, in greater or lesser numbers, is not this phenomenon an attempt on the part of nature to destroy the parasites at this phase of their life, through the action of the blood corpuscles, rather than a docile submission on the part of the corpuscles to the voracity of the germs? I am inclined to consider the ravages of the germs more extensive.

[1]FIG. 1. + 2000.

FIG 2. + 1000.

FIG. 3. + 1000.

FIG. 4. + 1000.

Indeed, the traces of these are plain in other parts than the blood, even in infectious Southern stock. In a sudden invasion of cattle unprotected by the immunity contracted South, or by artificial inoculation, the parasites develop with wonderful rapidity in many organs, which become impaired, and thus the nourishment on which the blood depends for rejuvenation becomes improper before the germs have overcome the blood corpuscles. The microbes seem to win the fight, because other parts of the body had

[1] For description of figures see page 454.

been previously weakened. We made some experiments to solve this problem. 1st, we produced Texas fever by *feeding* virus; 2d, by inoculation *subcutaneously;* and 3rd, by *intraveinous* inoculation. In the first case the germs appeared in the blood in sufficient quantity to master it only at a late hour—after the liver and spleen, etc., had been invaded and much impaired in their physiological functions. In the second case the germs appeared in the blood in a few hours, in large quantities, but most of them disappeared shortly, and four or five days later only a few germs and a few diseased corpuscles could be found; on killing the subjects, however, they were found plentiful in the liver and other organs. In the third case, in giving a moderate dose the animals became violently ill, and could scarcely rise; the blood had more germs than corpuscles; many of these perished, but eventually the germs became scarcer and scarcer, the distorted corpuscles also disappeared, and yet the subjects, when killed after they had sufficienly recovered to regain their feet and eat, their livers and some other organs still harbored millions.

Like Billings, I have found the germs motile but only in certain forms. In drop cultures, as well as in infected fluids or pulp from diseased subjects purposely killed, I have found this to be the case. They roll on themselves, change place under the microscope, advance, bend to and fro, try and shake themselves loose from their capsules, etc., etc.

The large forms in old bile sometimes show a slight waving motion. The spherical, ovoid forms and short rods, are often in zoöglea, both in fluids and in the liver. When such zoöglea are in drop cultures there may be a kind of slow bending, slow shaking motion on the part of some of the organisms to free themselves from the mass. The same thing is noticeable in cases of new cells trying to free themselves from the surrounding gelatinous matter of mother cells. A flagellum has been noticed at the extremities of some germs highly magnified. That appendage is perhaps the motor apparatus.

Before closing this article I sincerely ask my readers to kindly examine all the illustrations and read their explanations. They have much significance concerning the nature of this micro-organism. As stated, we are still engaged in studies to sift the biology of the forms mentioned in connection with our investigations.

Next year we hope to write in a more positive and more scientific line.

DEDUCTIONS.

1st. That the germ of Texas fever is susceptible to many changes during its vegetation.

2d. That the spherical, ovoid, and other forms, which several authors have seen, represent so many different periods in the life cycle of the parasite, or were different appearances due to different staining, including or excluding the envelope, and none of them separately could be sufficient to prove the identity and form a complete history establishing positively the discovery of the true nature of this micro-organism.

3d. That in the more mature stage the germs vary in size; some having measured 4 and 5$\mu$ in length, and 1.7$\mu$ in width; and that in the transitory ovoid stage, the dimensions may vary from 2.5$\mu$ in length, by 1.5$\mu$ in width, to .3$\mu$ by 1.5$\mu$.

4th. That probably the microbe passes only part of its existence in the animal body, and completes it in the outer world.

5th. That the modes of reproduction, the nature of its growth, and its various forms studied in its tissus, fluids and cultures, indicate an organism of the baccillar class, though possibly the adult stage in the outer world is akin to the form named cladothrix.

ARTIFICIAL CULTIVATION OF THE GERM OF TEXAS FEVER.

It is not my purpose to enter here into any scientific explanation that could not benefit those for whom this bulletin is written. I hope to give more details later with the biology of the microbe.

For the benefit of those unacquainted in the premises, I will say that artificial cultivation of germs means simply a process by which they may be reproduced at will in vases, test tubes, or dishes, just as seeds may be sown and plants reproduced and grown at will in a hot-house, at any time and during any season, even though the place of cultivation be foreign to the natural habitation of the plant. The disease germs are generally considered little plants, and artificial cultivation is one mode to grow them. When an animal or man is ill from any malady due to germs, it means that the germs in question are growing in that individual at the expense of the tissues, blood, etc. In cultivating disease germs, one substitutes an artificial nourishing media for the animal or other natural food.

No part of our work has offered more and greater difficulties than the artificial culture of the germ of Texas fever. Beef broth peptonized, solidified with agar-agar or gelatine, or in a liquid state ; blood serum, egg albumen (Tarchinoff's), amniotic water, artificial lymph, liver broth, potato, and several other media have been tried. We have found that they develop best in a mixture of artificial lymph that I prepared, with liver broth which Mr. Evans succeeded in keeping in a semi-solid and transparent state.

Pure cultures can be obtained from the liver, spleen, kidneys, etc., of *Southern cattle* of the *infectious districts*, or from their *calves before* or *after birth*. There need be *no post-mortem history about them*. Simply go South, kill the subjects, and with the greatest care possible inoculate your cultures by proper and safe bacterial methods, or take the germs from such organs of a Northern animal suffering from the fever. Or, if you prefer, you may find them but *less virulent* in the urine and fæces.

Pure cultures can also be obtained from the blood of such cases, but more rarely, and with greater difficulty. At some periods the germs seem to have little activity in the blood, and a particle of blood on a needle, thrust into a gelatinous mass, does not always cause a growth. However, we have succeeded in getting pure and inoculable cultures, by making an incision in the jugular vein, or carotid artery, inserting the point of a specially contrived test tube, breaking it inside of the vessel and allowing a drop or more to run into the tube and mix with the semi-fluid culture media. Cultures from blood have developed the *same forms* as the germs found in the liver and other organs, or found present in cultures therefrom.

### TRANSMISSION OF TEXAS FEVER.

As may have been gleaned from the preceding pages, there are many sources of contamination in this disease. Waters, soils, manures from the South, urine, bile, liver, spleen, kidneys, etc., of infectious Southern stock have been mentioned as being capable of conveying the fever. That is not all; we have found the parasites also in *ticks bloated with blood of infectious cattle*. So this must be added to the list of sources. But from all these origins there seems only one principal mode of infection in the natural condition of life, that is, *by ingestion*. I mean that the cattle swallow the germs and thus get the disease. It may be possible that germs deposited in dusty roads, or mixed with the dust raised by the winds, be inhaled, breathed by susceptible stock, and the malady thus induced, but such is certainly not the usual way.

These facts will satisfy, at least in part, the many opinions on the question at hand, and perhaps will help to clear the obscurity prevailing in all minds, and which I presume caused Dr. Theobald Smith to write as follows in his article already quoted: "As to the external character of the disease we have *still to learn how* Southern cattle carry the disease germs while they themselves are immune; how the germs multiply on the pasture and *how* they enter the susceptible organisms of the Northern cattle, and *whether or not they are* eliminated from the diseased body to become a *fresh centre of infection*."[1]

At this writing, I would not venture to give details as to the actual vegetation of the germs on the grounds, although I might be pardoned an opinion based on many laboratory cultures and some observations of other kinds. But as we are engaged in researches in that line I leave the point for another time. Sufficient it is to say, that germs excreted from off the body of Southern stock are *not at once* capable of much damage, but must first vegetate, thereby becoming virulent, whilst the virus from tissues, for instance, is more readily inoculable. Virus excreted by diseased Northern cattle is still weaker than from the Southerners, and becomes virulent outside after a longer time.

Texas fever is transmissible not only from Southern stock to susceptible Northern cattle, but under favorable circumstances is inoculable *between Northern natives*, although in the ordinary course of things *in our climate* transmission does not occur. We have inoculated native Missouri cattle with spleen and liver pulp from *other diseased natives* and produced typical cases of Texas fever, but it took large doses of virus. The rapidity of the course of the malady depends much on the origin and age of the virus. It was more rapid from old pulp kept in warmth and properly

[1] The italics are mine. Our experiments furnish answers to the Doctor's remarks, in part at least. P. P.

preserved than it was from virus of fresh matter, and it seems impossible to cause severe *Texas* fever with *fresh* urine, whilst the same exposed to warmth awhile becomes dangerous. We have succeeded, also, though with great difficulty, to induce the disease in sheep, guinea pigs, white mice, white rats, and very rarely rabbits, kittens, and swine. The germs may be reproduced by inoculation of liver and spleen pulp in any of these subjects, but the quantity must be large and the gross typical spleen lesions are not always to be found. Indeed, the *typical* spleen enlargement and softening, and the bloody urine in a case of Texas fever produced by inoculation are not always present, *nor* are they *always* present in ordinary accidental cases in cattle. It depends largely on the virulence of the matter inoculated, the atmospheric temperature, the susceptibility of the subject; and the virulence depends on the generation of, and the influences on, the germs outside, after their passage through one animal body, and before their reception by another individual. The very act of cattle eating fresh germs that have *just* been deposited on the ground by a Texas cow, for instance, is not necessarily followed by perceptible fever. We have tested this, and found that both on the grounds and in artificial cultures the first generation (or generations perhaps) coming from virus just excreted from the animal body are not dangerous. It seems that the parasite has a *resting* period after being thrown out, during which its virulence is very mild; and then the extreme virulence will come after several weeks, if the germs receive sufficient warmth and moisture. We have seen in this writing that native Missouri cattle exposed with Southern stock immediately on their arrival here, in a pen of a few square feet, where they must swallow the parasites while eating, did not show fever before about a month, and in cases where those pens were shaded it took longer still. In those cases the germs were brought here by Southern cattle and had to recuperate on our soil; it is the same thing when the disease is brought accidentally anywhere. Now, in cattle exposed in *Arkansas* and *Texas*, where the germs are *always* on the grounds, and consequently are already virulent, the animals die within an average of *twelve days* from date of landing, during the warm seasons. Reports from Drs. Dinwiddie, of Arkansas, and Francis, of Texas, show that our unprotected stock exposed there died within ten to fifteen days. Thus it takes once or twice as long for our native Northern stock to die of the fever when the germs are brought to *our* pastures by Southern cattle and dropped with manure and urine, as it takes when exposed to them on the *Southern soil itself*, where the parasites receive almost constant warmth and sufficient moisture, and are, therefore, always virulent. Germs artificially cultivated give results on the same principle.

Investigators who have pursued researches concerning Texas fever seem to have expected, by inoculation, always the typical lesions of the malady, as usually found when it originates by accident and runs an acute severe course. This is surely an unreasonable demand. One must allow for degrees in this disease as in any other. As stated above, the softness of the spleen and the bloody urine are not always present in accidental or purposely induced cases. It is simply the same malady with a lesser or greater degree of lesions. This is not the place to discuss this pathological question, however. I merely desire to draw the attention of the readers to the fact that in producing Texas fever by inoculation, if only mild lesions are produced, providing that they are the same in character as are those of a typical case, that they are produced by the same germ, that the same germs are found in both, that the cultivated germs of both cases are alike in forms and mode of vegetation, that inoculations practiced with germs of animals rendered ill by inoculation cause again the growth of similar germs and the advent of lesions of identical character, the proof is *sufficient*. In inoculating Texas fever one must always remember the peculiarities of its virus, its many forms, weak one day, stronger later on, etc.

The following tabulation of part of our inoculations speaks for itself. It is selected because of the variety of the virus used and the many sources. All the inoculations not otherwise specified were hypodermic and mostly in the tail.

Inoculation of any other animal than cattle has been found difficult. were made in the course of our experiments. Still the following tabulation for our purpose.

INOCULATIONS OF CATTLE

| Date of Inoculation. | Animals Inoculated. | Kind of Virus and Record Marks. | Average fever within 5 days. | Results. |
|---|---|---|---|---|
| 1889. | | | | |
| June 29.... | Red steer................ | Vaccine from urine of Texan | 103°, F. | Sent to Arkansas, lived.. |
| " 29.... | Red heifer............... | Vac. from manure of Texan | 103 1-5 | " " " .. |
| July 6..... | Red hornless heifer....... | Germs from ticks............ | 102 3-5 | Contracted fever in Ark.. |
| " 20.... | Small red heifer.......... | Vac. from spleen of native... | 103 2 5 | Exposed to fever, lived.. |
| " 20.... | Spayed heifer............ | " " " " ... | 103 | " " " .. |
| " 26.... | Roan heifer.............. | Culture I 4................... | 103½ | " " " .. |
| " 26.... | Red heifer............... | Culture L 3.................. | 103 3-5 | " " " .. |
| " 26.... | Red heifer............... | Vac. from liver of Ark. cow.. | 103 | " " " .. |
| Aug. 9..... | Heifer.................... | Strong virus from liver Texan | 104 1-5 | Died on car.............. |
| " 15.... | Long-horned cow........ | FED potato culture U 6....... | 106 | Died Aug. 22.............. |
| " 16.... | Small spotted heifer...... | Intraveinous inoculation U6 | .......... | Died Aug. 16.............. |
| " 16.... | Heifer, right horn broken | Native liver and spleen pulp | 105 3-5 | Died Aug. 24.............. |
| Sept. 5..... | Big-jaw steer............ | Culture BT3................. | 103 | Sick; killed Sept. 20..... |
| " 5..... | Old cow.................. | Culture U6'.................. | 103 1·5 | Recovered................ |
| Oct. 3..... | Cow No. 5................ | Culture 9½ IN VEIN.......... | 104 | Exceedingly ill; recover'd |
| " 16, 19,23 | Forty-one bulls........... | Mild and strong vaccine...... | 104 | Some stagger'd; 40 expos'd Indian Ter'y; 39 lived..... |
| Dec. 28.... | Heifer.................... | Vaccine virus weak ABM.... | 103 | Slightly ill............... |
| " 31.... | Calf, four months......... | Culture XY'A................ | 102 1-5 | Very slightly ill.......... |
| " 16,21,23 | Sixty-five head cattle..... | Very strong vac. large doses.. | 105 | All ill; 5 deaths.......... |
| " 24.... | Thirty head Herefords... | Graduated vaccine........... | 103 | Sent to Texas and lived.. |
| 1890. | | | | |
| Jan 17.... | Red roan heifer.......... | Cult. AM' VEINOUS inoculat'n | 102 3-5 | Very sick................. |
| " 17.... | Little red steer........... | Cult. AM'.................... | 103 | Exceedingly sick; killed |
| " 17.... | Old Cow.................. | Cul . AM' in VEIN........... | 103 1-5 | " " staggers |
| Feb. 13.... | Heifer.................... | Old manure moistened, etc.. | 103 1-5 | Weakly.................. |
| " 13.... | Stall No. 3 heifer........ | Cult. P. A'.................. | 103 | Ill and weak............. |
| " 13.... | Stall No. 4 steer.......... | Cult. FA3 from ticks......... | 103 1-5 | Weakly.................. |
| " 13.... | Steer..................... | Granules from liver of South'r | 102 4-5 | Slightly ill............... |
| Mar. 20.... | Cow...................... | Granules from bile of native. | 102 4 5 | Indisposed............... |

It always took large doses. Many fruitless, a few successful attempts does not give all that we have on record, but shows a sufficient variety

AT LABORATORY, ETC.

---

Microscopic Revelations, Post-Mortem Appearances and Remarks.

---

Lesions of Texas fever. Had previously in blood, germs produced by inoculation from the ticks

Germs found in the blood taken from the ear. Corpuscles mildly but typically impaired.

Found the germs in drops of blood ; corpuscles distorted.

Mr. Hampton pronounced it Texas fever. It was produced by inoculation.

Typical case of Texas Fever ; soft spleen, bloody urine and the germs.

In a few hours the blood was mastered by the germs. SAME CULTURE that caused preceding death.

Typical case of Texas fever. The germs present.

Mild lesions of Texas fever. The germs present.

The blood presented the germs, and the corpuscles were typically diseased.

Blood full of Texas fever germs for several days—gradually disappeared.

Cattle of J. J. McAlester.
Germs in the blood as inoculated and the blood mildly modified, as in Texas fever.

Germs in blood and corpuscles impaired, as in Texas fever.

Germs present in blood, which is clearly though mildly affected.

Characteristic cases of Texas fever.

Cattle of Mr. C. D. Foote.

Typical germs and lesions of Texas fever in blood.

Germs of Texas fever in blood, bile, liver, etc. ; lesions in tissues mild, but characteristic.

Germs found in blood, which is much affected, typically.

The blood and corpuscles show the germs of Texas fever.

Killed within three days, and germs of Texas fever found. Typically distributed in liver, spleen, blood.

Blood analyzed and found to contain the germs, as inoculated. Corpuscles distorted.

Texas fever germs found in blood.

Corpuscles distorted, etc. Germs in blood and affecting the corpuscles.

---

### INOCULATIONS OF OTHER ANIMALS THAN CATTLE.

| Date of Inoculation. | Animals Inoculated. | Kind of Virus Used and Record Marks. | Results. | Microscopic and Post-Mortem Appearances and Remarks. |
|---|---|---|---|---|
| 1889. | | | | |
| July 10....... | Gopher.......... | Liver of Ark. steer....... | Seriously ill: staggers... | Germs developed locally, then generalized, subject recovered. |
| July 10....... | Black Guinea Pig | " " " ....... | Died Aug. 12............. | Germs of Texas fever found in blood, liver, spleen, etc. |
| July 11....... | Yellow Guinea Pig | Cult. A3 of Texas cattle urine. | " " "............. | Germs similar as found in preceding cases and in same organs. |
| Aug. 2....... | White Guinea Pig | Plv. spleen native cattle.. | Apparently ill........... | Recovered. |
| Aug. 15...... | Black Guinea Pig | Culture U3.............. | Died —— — ............. | Post-mortem, unfortunately, missed until too late. |
| Aug. 16 to Sept. 3 .... | Thirteen different Rabbits......... | Different fresh and old cultures................ | Some slightly ill; none seriously................ | Occasionally corpuscles of the blood found diseased; somewhat crenelated. |
| Sept. 4....... | Rabbit in cage 11 | Large dose cult. BO3..... | Died Sept. 7th........... | Found germs of Texas fever in liver, spleen and blood. |
| Sept. 5....... | Guidea Pig cage 2 | Culture D2.............. | Slightly ill............... | Found germs of Texas fever in blood. |
| Sept. 9....... | 6 mos. ordinary pig (swine)..... | Liver of native with Texas fever............ | Died Sept. 13th.......... | Lesions in liver and blood as those of Texas fever; same germs; they produced good cultures. See figures. |
| Oct. 1........ | Black Kitten...... | Cult. 9½ from native cow | Died Oct. 2d............. | Germs found in blood, liver, etc., as those of Texas fever. |
| Oct. 11....... | 2 small White Mice | Cult. CF4................ | Died Oct. 13th........... | Liver enlarged; blood, liver and spleen had Texas fever germs. |
| Oct. 18....... | 2 White Mice..... | Cult. D″′................ | Exceedingly sick; 1 killed Oct. 19, other died Oct. 20 | Germs and lesions similar to those of Texas fever; spleen not enlarged but gorged slightly. |
| Oct. 18....... | 2 White Mice..... | Cult. D13................ | One died Oct. 19th........ | Same lesions and germs as in preceding case. |
| Dec. 28....... | 3 White Rats..... | Cults. ABM′, X″ A., 9½ A | All slightly ill........... | Germs found in their blood as in Texas fever. |
| Feb. 13....... | 2 Sheep.......... | PA2 and PA3 in thigh.... | Decided lameness and illness. | Germs found in their blood as in Texas fever. |
| Feb. 13....... | 2 White Rats..... | " " in tail...... | One died Feb. 15th ....... | Both have germs of Texas fever. |

In all the inoculations mentioned above, it was possible to identify the germs satisfactorily, and then make from various specimens, cultures that became virulent and produced *in cattle* the same germs, the congestion of the liver, the distorted blood corpuscles, etc., as occur in Texas fever.

DEDUCTIONS.

1st. That susceptible cattle contract the disease generally by swallowing the germs thrown out with urine, manure, etc., and that the breath does not cause it.

2d. That the germs after passing through the animal body are not ready to cause disease, but take some time to become deadly, consequently the germs spread in the usual way on Northern grounds must first recuperate virulence, *unless* they were brought from the South in *old manure and old urine* adhering to the feet.

3d That ticks full of blood from infectious Southern cattle may, it seems, scatter the germs on our lands, though ticks *of themselves cannot* convey the disease.

4th. That the disease is inoculable from native to native, and would be transmitted between them if our warm seasons were extended enough, and if our frosts were lighter.

5th. That the malady is inoculable in a mild form to other animals than cattle, such as sheep, pigs, guinea pigs, white rats, white mice, etc.

6th. That in all animals inoculated, the germs of yellow fever, and the most truly characteristic lesions that they cause, are reproduced in a more or less pronounced form.

THE PRINCIPLE OF IMMUNITY—HOW TEXAS CATTLE CARRY THE GERMS OF TEXAS FEVER AND YET REMAIN APPARENTLY HEALTHY. VACCINATION.

There are many theories advanced concerning the principle underlying immunity conferred by inoculation, vaccination, or an attack of disease. Smallpox is prevented by one attack, or by vaccination with cowpox,[1] during the existence of which the system is rendered proof against smallpox for a *certain period;* charbon is prevented *for a certain period* by one attack accidentally contracted, or purposely inoculated in a mild form; black leg is prevented just the same way, and so are several other affections of a specific character. Texas fever we found to be preventable on the same line. As mentioned, immunity, however, exists in any disease only for a time. Smallpox for several years, black

[1] Some renowned authorities claim that cow pox and horse pox are identical.

leg for several years, charbon for a few months, Texas fever for a few months, etc., etc. In our experiments we found, as my readers have learned, that the Texas fever germs are always living in the bodies of the infectious Southern cattle, and yet these remain apparently healthy. Thus, whilst the germs grow in them, they are proof against an acute attack of the disease. But if infectious Southern cattle are kept *North*, say in Missouri, for about a year, and then exposed to Texas fever again, they will contract the disease and may die. So they have immunity, or in other words are proof against the disease only for a short period, a few months or a year at most. This has been verified by actual shipments near Sturgeon, Mo., three years ago, and by others throughout the State. The fact that Southern cattle which are proof against the disease have, nevertheless, the germs of it constantly growing in their systems, shatters a few theories of a general character, attempting to explain by what phenomenon the body actually becomes vested with power to resist disease. In Texas fever as, perhaps, in several infectious diseases, it seems only a *question of tolerance*. The body becomes accustomed to the action of the germs and its products just as one becomes accustomed to take morphine, or to the climatic influences of fever and ague.

Now, as to how Southern cattle bring us disease, whilst they seem free, it is very simple. They have been naturally inoculated or vaccinated from their mother's womb before birth, and then have been subject to the influence of the germs all their lives. Thus they can just as safely carry the germs on them, in their bowels, etc., as a man, having had smallpox, or having been vaccinated, may carry the germs of smallpox on his person in any way, shape or form. Indeed, he might swallow them with impunity to himself, and yet might distribute the virus and cause smallpox in others. Now, concerning the transmission between natives; that rests solely on two conditions: the nature of the germs, and the nature of the climate. It has been seen that the germs on Southern soil, where it is always warm, more or less, are always deadly except during cold times, and that cattle exposed there die within ten or twelve days. The same germs gathered on Southern soil and brought here in a glass jar, say, have the same effects. But if Southern cattle bring us the germs in

*their bodies, i. e.*, in their bowels and urine, the condition is changed. In such cases the germs deposited on our grounds remain harmless between one and two months at least. During that time, exposed to sun heat, and sufficient moisture, the virulence is regained, and *then* exposed susceptible cattle sicken and die. Now, when our natives get the fever, they deposit the germs on the ground just as the Southern stock, but before the germs can gain virulence the cold and frosty season of the North *is on them*, and modifies and even destroys their activity. Some experiments, not given in detail in this bulletin, show, as all observations do, that *cool weather* retards the *growth* of the germs, that *freezing point* for *several hours* intercepts the *vegetation* to adult forms, and *long frosts* modify these even to destruction. The reason, therefore, that in the ordinary course of things native cattle suffering from Texas fever do not transmit the disease to others, is simply because the duration of our warm season is not long enough to allow the germs to grow in virulence, after passing through the animal body. By inoculation and cultivation of germs from such stock we found this to be the truth, and those of my readers who have had the patience to read this carefully have perhaps already come to this conclusion themselves.

### CONCLUSIONS.

The conclusions that we have arrived at from the facts developed by *all our experiments* and researches are given here in direct reply to each proposition that we formulated when we began our work.

1st. The Texas fever germs may be found in some surface soils, grasses and pond waters of the infectious districts of the Southern states.

2d. The virus is found in the liver, spleen, lymph glands, kidneys, blood, bile, urine and fæces (fresh droppings of manure), and is transferred to the North chiefly by the urine and manure.

3d. Ticks and the feet of cattle are capable of carrying the germs to distant lands.

4th. The period of *incubation, i. e.*, the lapse of time *between* the *moment* that germs are taken into the *body* by susceptible cattle and the *appearance* of the disease, is between eight and twelve days only. Cattle may be *exposed longer* and not become affected, but this depends on the weakness of the germs in conditions demonstrated by our field tests and inoculations.

5th. So far, experiments indicate that about 30 *days after leaving the Southern infectious soil*, the Southern cattle are not dangerous ; consequently, if they were kept that long in quarantine North of the fever line, they could then be safely scattered among Northern stock. More experiments are necessary on this point.

6th. The cheapest mode that we know now to disinfect cars and yards, is by quicklime, corrosive sublimate solution, or steam; but here again we mean to experiment further. Chloride of lim - has little value except to impart a pleasanter odor.

7th. The means to disinfect Southern cattle alive, and render them harmless before shipping North, is not settled, and we shall experiment with that end in view this year.

8th. Inoculation was at first very unsuccessful, but afterwards proved beneficial. Properly inoculated cattle were shipped and exposed South with *little or no* damage. More experiments are needed on this point.

9th. Other animals than cattle may, under certain conditions, when shipped by rapid transit, bring Texas fever North, and one good observation indicates that a shipment of horses has done so in the State of Missouri.

10th. During favorable weather, virus spread in the North with fresh manure and urine directly from Southern cattle, becomes virulent in about 30 days (and perhaps occasionally less) during the warm months, and remains virulent until decidedly cool weather.

11th. Under the circumstances explained in this bulletin, Northern cattle suffering from Texas fever may communicate the disease to other Northern natives, though this cannot occur in the ordinary course of things in Missouri, or anywhere North, because cold weather arrives too soon to allow the vegetation of the germ to virulent maturity.

12th. Calves born South become inoculated or receive the germs of Texas fever directly from their mothers before birth, and then continue to resist the germs, first, because of this *natural* inoculation, and second, because of receiving the virus gradually, perhaps in the milk, and then when they begin to eat or nibble on grass.

### EXPLANATION OF THE FIGURES.

Fig. 1. The microbe of Texas fever at various stages of its development, from a shaken liver-broth culture pure, one day old (that became inoculable). Origin from the blood of a native suffering from Texas fever.

Letters a, d, h, point to the SMALL OVOID STAGE described by Frank S. Billings as the adult form; the letters b, i, g, present ovoid forms elongating and spots within; the letters o, c, as well as the b pointing to a small circle to the left of the figure, show the cocoid appearance, or perhaps the same forms as the preceding, standing on end, and thus presenting a view of the top of one extremity; m, fine new forms to develop in the ovoid represented by a, b, h; f, r, germs sufficiently developed to show by dots, what seems at this stage to be the ends of new ovoid forms within; p, still further development of a mother cell showing two fine ovoid bodies within, which are to be freed, and appear then as shown by letters m, and later a, b, etc., etc.; e, k, figure-8 germ—an appearance probably identical with Dr. Salmon's bacteria of Texas fever.

Fig. 2. Forms in a photograph of old BILE of a cow killed when suffering from Texas fever. Some may be crystals (?), but I think most represent the adult forms in the outer world. Some forms in such bile, not shown in the cut, had a flagellum, or prolongation at each end.

Fig. 3. Germs from a culture in beef broth; very well developed; proved inoculable.

Fig. 4. Germs from liver of HEALTHY Arkansas steer killed purposely; proved inoculable; furnished the culture represented in Fig. 3.

## Figures and Tables

**Fig. 1. f1:**
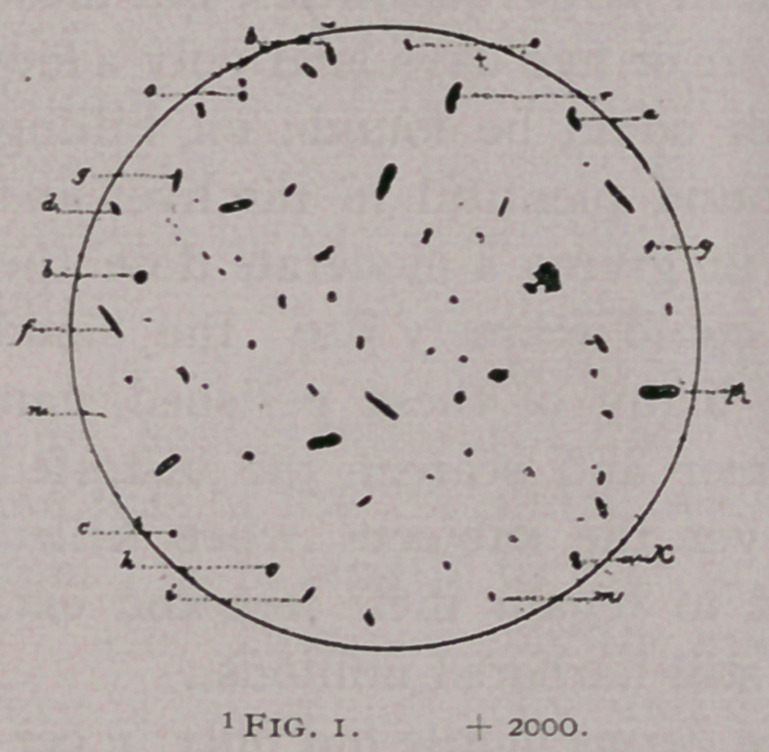


**Fig. 2. f2:**
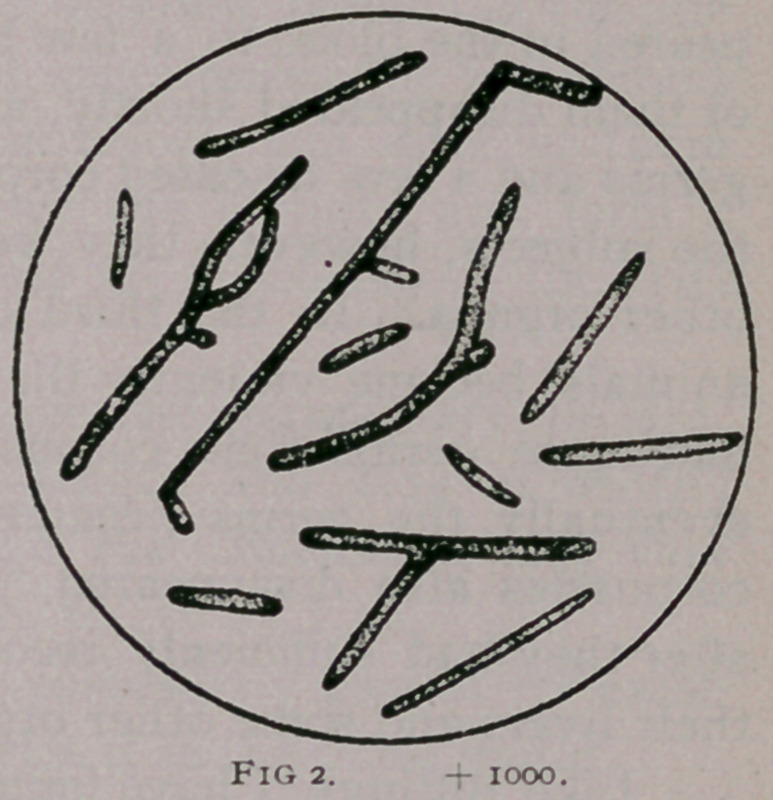


**Fig. 3. f3:**
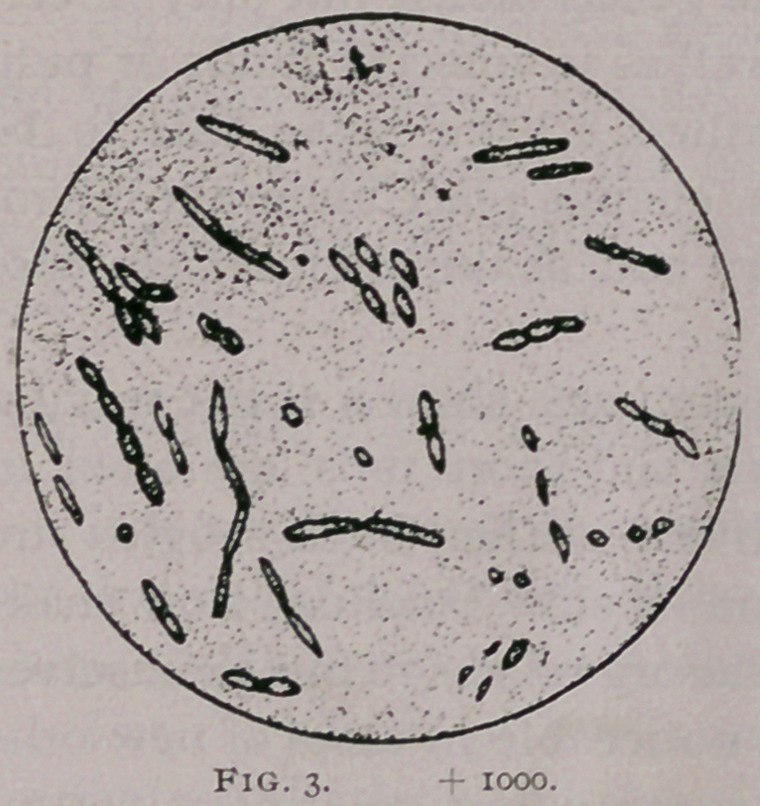


**Fig. 4. f4:**